# Is dynamic thiol/disulfide homeostasis associated with the prognosis of myelodysplastic syndrome?

**DOI:** 10.2478/jomb-2019-0050

**Published:** 2020-09-02

**Authors:** Ucar Mehmet Ali, Anıl Tombak, Simten Dagdas, Aydan Akdeniz, Funda Ceran, Salim Neselioglu, Ozcan Erel, Gulsum Ozet

**Affiliations:** 1 Mersin University, Faculty of Medicine, Department of Hematology, Mersin, Turkey; 2 Ankara Numune Training and Research Hospital, Department of Hematology, Ankara, Turkey; 3 Yıldırım Beyazıt University, Faculty of Medicine, Department of Hematology, Ankara, Turkey

**Keywords:** disulfide, mercaptan, myelodysplasia, oxidative stress, thiol, disulfid, merkaptan, mijelodisplazija, oksidativni stres, tiol

## Abstract

**Background:**

This study planned to investigate the relationship of dynamic thiol/disulfide homeostasis with the prognosis of myelodysplastic syndrome (MDS).

**Methods:**

80 patients who had been diagnosed with MDS between 2012 and 2017 and who were older than 18 were included in the study together with 80 healthy control subjects. The MDS diagnosis was confirmed using bone marrow aspiration-biopsy immunostaining. Dynamic thiol/disulfide homeostasis and ischemia-modified albumin (IMA) levels were examined.

**Results:**

The average IMA (0.71±0.08 vs. 0.67±0.09; p=0.002), median disulfide (18.0 vs. 11.6; p<0.001), median disulfide/native thiol (6 vs. 3; p<0.001), and median disulfide/total thiol (5.4 vs. 2.9; p<0.001) were found higher in the MDS patients compared to control group, and the median hemoglobin, median white blood cell count, median neutrophil count, median lymphocyte count, average native thiol (290.7±48.5 vs. 371.5±103.8; p<0.001), average total thiol (328.2±48.9 vs. 393±105.5; p<0.001), and average native thiol/total thiol (%) (88.3±4.3 vs. 94.2±2.1; p<0.001) were found to below. Risk factors such as collagen tissue disease (HR:9.17; p=0.005), MDS-EB-1 (HR:10.14; p=0.032), MDS-EB-2 (HR:18.2; p=0.043), and disulfide/native thiol (HR:1.17; p=0.023) were found as the independent predictors anticipating progression to acute myeloid leukemia. In the Cox regression model, risk factors such as age (HR:1.05; p=0.002), MDS-EB-1 (HR:12.58; p<0.001), MDS-EB-2 (HR:5.75; p=0.033), disulfide/native thiol (HR:1.14; p=0.040), and hemoglobin (HR:0.64; p=0.007) were found as predictors anticipating for mortality.

**Conclusions:**

We can argue that dynamic thiol/disulfide homeostasis could have significant effects on both the etiopathogenesis and the survival of patients with MDS, and it could be included in new prognostic scoring systems.

## Abstract (sr)

**Uvod:**

Plan ove studije je bio da istraži vezu dinamične tiol/disulfidne homeostaze i prognoze mijelodisplastičnog sindroma (MDS).

**Metode:**

U istraživanje je uključeno 80 pacijenata kojima je dijagnostifikovan MDS između 2012. i 2017, starijih od 18 godina, i 80 zdravih kontrolnih ispitanika. MDS dijagnoza je potvrđena imunološkim bojenjem koštane srži dobijene aspiracionom biopsijom. Ispitani su dinamična tiol/disulfidna homeostaza i nivoi albumina modifikovanog ishemijom (IMA).

**Rezultati:**

Otkriveno je da su vrednosti prosečnog IMA (0,71 ± 0,08 nasuprot 0,67 ± 0,09; p = 0,002), vrednost medijane disulfida (18,0 naspram 11,6; p < 0,001) i disulfid/nativniog tiola (6 naspram 3; p < 0,001) i medijane disulfid/ukupnog tiola (5,4 naspram 2,9; p < 0,001) veće kod bolesnika sa MDS-om u poređenju sa kontrolnom grupom. Takođe, otkrivene su i niske vrednosti medijane hemoglobina, belih krvnih zrnaca, neutrofila, limfocita, prosečnog nativnog tiola (290,7 ± 48,5 naspram 371,5 ± 103,8; p < 0,001), prosečnog ukupnog tiola (328,2 ± 48,9 u odnosu na 393 ± 105,5; p <0,001) i prosečnog nativnog tiola/ukupni tiol (%) (88,3 ± 4,3 prema 94,2 ± 2,1; p < 0,001). Faktori rizika poput bolesti kolagenskih tkiva (HR: 9,17; p = 0,005), MDS-EB-1 (HR: 10,14; p = 0,032), MDS-EB-2 (HR: 18,2; p = 0,043), i disulfid/nativni tiol (HR: 1,17; p = 0,023) su otkriveni kao nezavisni prediktori koji predviđaju napredovanje do akutne mijeloidne leukemije. Po Koksovom modelu regresije, faktori rizika kao što su starost (HR: 1,05; p = 0,002), MDS-EB-1 (HR: 12,58; p <0,001), MDS-EB-2 (HR: 5,75; p = 0,033), disulfid/nativni tiol (HR: 1,14; p = 0,040) i hemoglobin (HR: 0,64; p = 0,007) smatraju se prediktorima smrtnosti.

**Zaključak:**

Možemo tvrditi da bi dinamička tiol-disulfidna homeostaza mogla da ima značajne efekte i na etiopatogenezu i na preživljavanje pacijenata sa MDS-om i da bi mogla biti uključena u nove prognostičke skoring sisteme.

## Introduction

Myelodysplastic syndrome (MDS) is a heterogeneous clonal hemopoietic stem cell disease characterized by various levels of cytopenias and abnormal myeloid cell differentiation and maturation in addition to acute leukemia transformation risk. Although cytopenia (anemia, neutropenia, and thrombocytopenia) is seen in MDS patients, the bone marrow (BM) is often hypercellular and dysplastic, and the functions of the blood cells in circulation are disrupted. Although MDS could develop de novo, it could also develop secondary to previously administered chemotherapy and radiotherapy. All three of the erythrocytic, megakaryocytic, and granulocytic series in the BM could be affected. It is a stealthy disease generally developing in men, with increasing incidence rate as the age increases, and primarily seen above 65 years of age [1] [2].

There is no single factor responsible for MDS etiopathogenesis. Alkylating agents, benzene, ionizing radiation, and other toxic chemical agents causing deformations in the DNA structure have all been implicated in the etiopathogenesis. In addition, it is thought that increased oxidative stress contributes to DNA damage. The reactive oxygen species (ROS) produced in all aerobic cells are removed by antioxidants. Oxidative stress emerges with the disruption of the balance between reactive oxygen production and the antioxidant system. Reactive oxygen contributes to DNA damage with toxic materials in oxidative damage [3] [4]. Several enzymatic and non-enzymatic mechanisms are involved to protect the organism against the harmful effects of ROS [5]. One of these antioxidant molecules is thiol, and these molecules are compounds containing sulfhydryl groups, playing a critical role in preventing oxidative stress. The primary targets of ROS are the sulfur groups containing the amino acids of the proteins (cysteine, methionine). The thiol groups are oxidized with the ROS that form reversible disulfide bonds. This transformation is the first discovered marker of radical-mediated protein oxidation. The disulfide bonds formed in this way could be degraded to thiol groups again by several antioxidants, and thus the thiol/disulfide homeostasis is continued [6] [7] [8] [9].

Dynamic thiol/disulfide homeostasis plays an important role in intra-and intercellular signal transmission, the regulation of apoptotic pathways, enzyme activation and inhibition, and the regulation of factors related to the oxidant-antioxidant system and transcription [10]. It has been shown that anomalies in abnormal dynamic thiol/disulfide homeostasis are prominent in diseases such as malignities, Alzheimer's disease, hyperemesis gravidarum, Friedreich's ataxia, multiple sclerosis, diabetes mellitus, cardiovascular diseases, and rheumatoid arthritis [11]. However, we could not find any study on the relationship between dynamic thiol/disulfide homeostasis and MDS. Therefore, this study aimed at investigating the relationship of dynamic thiol/disulfide homeostasis with MDS prognosis.

## Materials and Methods

The study was conducted retrospectively with patients diagnosed with MDS in the Mersin University Internal Diseases Department, Hematology Section.

The study included 80 patients above 18 years of age diagnosed with MDS between 2012 and 2018, together with 80 healthy control subjects. Individuals were excluded from the study if they had acute or chronic liver diseases, rheumatologic diseases, or active infections; if they were using antioxidant substances or lipid-lowering drugs; or if they smoked, consumed alcohol, or used vitamin supplements.

In addition to the demographic information of the patients, their chronic diseases and accompanying comorbidities were recorded. The MDS diagnosis was confirmed using bone marrow (BM) aspirationbiopsy immunostaining. Tissue samples were evaluated in line with the 2008 World Health Organization classification. The International Prognosis Scoring System (IPSS) and the Revised International Prognosis Scoring System (R-IPSS) were used in prognostic scoring with the addition of cytogenetic analysis, the number of blasts in the BM, and the cytopenia level. In the IPSS and R-IPSS, all three variables are given a score from 0 to 2, and then the three values are added, and the IPSS and R-IPSS scores are obtained [12] [13].

This study was conducted in compliance with the principles of the Declaration of Helsinki and was approved by the Mersin University Faculty of Medicine's Ethics Committee (ethical committee decision date: 10.07.2019, 2019/300, no: 78017789/050.01.04/1102393)

### Biochemical Evaluation

To measure the ischemia-modified albumin (IMA) levels of all participants of the study, blood samples were taken from the antecubital vein between 08:00 and 10:00 a.m. following overnight fasting. The blood samples were centrifuged at 4000 rpm for 10 minutes, and the serum and plasma samples were separated. Serum samples were stored at -80 °C until all blood samples were collected.

Albumin was measured using the bromine cresol green method with a Hitachi Modular P800 autoanalyzer (Roche Diagnostic Corp., Indianapolis, IN, USA). The erythrocytes and thrombocytes were measured using the impedance (resistance) method, leukocytes were measured with the optical laser scattering method, and other hemogram parameters were measured using the Sysmex XE 2100 hematology analyzer (Roche Diagnostic). Hemoglobin was measured photometrically.

### IMA measurement

The IMA levels were measured with a serum ELISA kit. The results were expressed in ng/mL.

### Dynamic thiol/disulfide homeostasis

Blood samples were taken from patients when the MDS diagnosis was made to evaluate all serum thiol types and disulfide levels, and fasting blood samples were also taken into sampling tubes from the control subjects. After venous blood samples were taken, they were stored for 20 minutes for clotting, and the serum was separated by centrifugation at 1600 rpm for 10 minutes. Samples were then stored at -80 °C until analysis. All samples were processed in the same manner. After the study was finished, all samples were thawed, and tests for thiol/disulfide homeostasis were conducted. The thiol/disulfide homeostasis tests were performed using a new automatic and spectrophotometric method developed by Erel and Neselioglu [10].

First, the free functional thiol groups were extracted by the degradation of the disulfide bonds using sodium borohydride. The unused reduced sodium borohydride was suppressed with formaldehyde to prevent the decrease of 5.5-dithiobis-(2-nitrobenzoic acid) (DTNB). After the reaction with DTNB, the total thiol groups, including both reduced and natural thiol groups, were determined. Dynamic disulfide amount was obtained by the division of the variance between the total and the natural thiol. The disulfide/total thiol, disulfide/native thiol, and native thiol/total thiol levels were calculated as percentages.

### Statistical Analysis

Statistical analyses were conducted using SPSS 20 for Windows (IBM Corp., Armonk, NY, USA) and MedCalc 11.4.2 (MedCalc Software, Mariakerke, Belgium) software. The normal distribution of the data was evaluated using the Kolmogorov-Smirnov test. The normally distributed numerical variables were presented as median ± standard deviation, and data that were not normally distributed were presented as median (min-max). Categorical variables were presented as number and percentage. For two-group comparisons of independent samples in the analysis of the numerical variables with normal distribution, the t-test was used, and for those that did not present normal distribution the Mann-Whitney U test was used. In the comparison of three or more groups, ANOVA (post hoc: Bonferroni test) was used for independent samples in the analysis of the numerical variables with normal distribution, and the Kruskal-Wallis H (post hoc: Dunn test) was used for those without normal distribution. The chi-square test and Fisher exact chi-square test were used for the comparison of categorical values. The relationships between oxidative stress parameters and numerical variables were analyzed using Spearman correlation analysis. Univariate Cox regression analysis was used in determining the potential factors affecting acute myeloid leukemia (AML) transformation and mortality, and the independent risk factors were defined by adding the previously determined potential risk factors into the multivariate Cox regression model. The prediction values pertaining to the oxidative stress parameters were analyzed with ROC curves. p<0.05 was acknowledged as significant in the statistical analyses.

## Results

The clinical demographics and laboratory findings pertaining to the study population are presented in detail in Table 1. The average IMA (0.71±0.08 vs. 0.67±0.09; p=0.002), median disulfide (18.0 vs. 11.6; p<0.001), median disulfide/native thiol (6 vs. 3; p<0.001), and median disulfide/total thiol (5.4 vs. 2.9; p<0.001) were found higher in the MDS patients, while the average hemoglobin (9.7±1.8 vs. 15.1±0.6; p<0.001), median white blood cell (WBC) count (3800 vs. 7500; p<0.001), median neutrophil count (2000 vs. 4800; p<0.001), median lymphocyte count (1470 vs. 2000; p<0.001), average native thiol (290.7±48.5 vs. 371.5±103.8; p<0.001), average total thiol (328.2±48.9 vs. 393± 105.5; p<0.001), and average native thiol/total thiol (%) (88.3±4.3 vs. 94.2±2.1; p<0.001) were found to be lower.

**Table 1:** Clinical and laboratory findings of the study population *Parameters were expressed as mean ± SD and median (interquartile range). *P<0.05 is considered significant for statistical analyses. *Abbreviations*: IMA: Ischemia Modified Albumin, WBC: White Blood Cell

Variables	Total n=160 MDS n=80	Control n= 80	p	
Age (Year)	54.5±17.4	66.6±11.5	64.4±13.5	0.268
Gender (%)				
Female	51(31.9)	28(35.0)	23(28.8)	
Male	109(68.1)	52(65.0)	57(71.3)	0.396
Albumin (g/L)	43±3	4.2±0.3	4.3±0.2	0.443
IMA, (ng/mL)	0.69±0.09	0.71±0.08	0.67±0.09	0.002*
Natıve thiol (μmol/L)	331.1±90.3	290.7±48.5	371.5±103.8	<0.001*
Total thıol (μmol/L)	360.6±88.2	328.2±48.9	393±105.5	<0.001*
Dısulfıde (μmol/L)	13.1(3–32.6)	18(10.7–32.6)	11.6(3–14)	<0.001*
SS/SH (%)	4(0.7–19.4)	6(3.6 –19.4)	3(0.7–10.6)	<0.001*
SS/TT (%)	3.7(0.7–14)	5.4(3.4–14)	2.9(0.7– 8.8)	<0.001*
SH/TT (%)	91.2±4.5	88.3±4.3	94.2±2.1	<0.001*
WBC (x103 μL)	6200(1300–22000)	3800(1300–22000)	7500(3300 –16900)	<0.001*
Neutrophile (x10^3^ μL)	3300(200–13600)	2000(200–9450)	4800(1300–13600)	<0.001*
Lymphocyte (x10^3^ μL)	1800(430–4200)	1470(430–3320)	2000(500–4200)	<0.001*
Hemoglobin (x10^3^ g/L)	12.4±3.1	9.7±1.8	15.1±0.6	<0.001*
Platelet (x10^3^ μL)	249.9(10–652.6)	98(10–626)	356.6(146.5–652.6)	<0.001

The comorbidity distribution of the MDS patients included hypertension in 67.5% (n: 52), cardiac disease in 32.5% (n: 26), thyroid disease in 13.8% (n: 11), chronic obstructive pulmonary disease (COPD) in 13.8% (n: 11), renal disease in 10% (n: 8), cerebrovascular disease (CVD) in 5% (n: 4), and collagen tissue disease in 5% (n: 4).

It was found that 6.3% of the MDS patients had solid MDS, 31.3% had MDS with ring sideroblasts (MDS-RS), 20% had MDS with multilineage dysplasia (MDS-MLD), 25% had MDS with excess blasts-1 (MDS-EB-1), 8.8% had MDS with excess blasts-2 (MDS-EB-2), 2.5% had MDS with isolated del(5q), and 6.3% had MDS of unclassifiable type. In the MDS-EB-1 patients, the median disulfide level, disulfide/native thiol ratio, and disulfide/total thiol ratio were higher compared to patients with the solid, MDS-RS, and MDS-MLD types while the native thiol/total thiol ratio was lower. In MDS-EB-1 patients, the median disulfide level, disulfide/native thiol ratio, and disulfide/total thiol ratio were found lower when compared to the MDS-EB-2, del(5q), and unclassifiable types and the native thiol/total thiol ratio was higher. In MDS patients with Int-2 IPSS scores, the average native thiol level, average total thiol level, and disulfide/total thiol ratio were found lower when compared to patients with low and Int-1 IPSS scores, and the median disulfide level, disulfide/native thiol ratio, and disulfide/total thiol ratio were found higher. In MDS patients with Int-1 and low IPSS scores, the oxidative stress parameters did not show any significant differences. In MDS patients with high R-IPSS scores, the average native thiol level, average total thiol level, and disulfide/total thiol ratio were found lower compared to those of patients with other IPSS scores, and the median disulfide level, disulfide/native thiol ratio, and disulfide/total thiol ratio were found higher. The oxidative stress parameters did not show any significant differences in patients with other R-IPSS scores. No significant correlation could be found between the cytogenetic parameters and oxidative stress parameters. The disulfide level, disulfide/native thiol ratio, and disulfide/total thiol ratio were found lower in patients with EPO compared to patients without, and no significant correlation could be found between other oxidative stress parameters (Table 2).

**Table 2:** Distribution of oxidative stress parameters according to clinical features in MDS patients Parameters were expressed as mean ± SD and median (interquartile range), *P < 0.05 is considered significant for statistical analysis. *Abbreviations*: MDS, Myelodysplastic Syndrome; IPSS, International Prognosis Scoring System; R-IPSS, Revised International Prognosis ScoringSystem.

Variables	n (%)	Natıve Thıol (μmol/L)	p	Total Thıol (μmol/L)	p	Dısulfıde (μmol/)	p	SS/S (%)	p	SS/T (%)	p	SH/TT (%)	p
MDS type													
MDS-Sld	5(6.3)	307.3±19.4		336.7±19.5		14.3(11.5–18.3)		4.3(3.9–5.9)		4.0(3.6–5.2)		91.3±1.6	
MDS-Rs	25(31.3)	297.2±45.1		330.8±48.2		14.7(10.7–29.6)		4.6(3.6–10.6)		4.7(3.4–8.8)		89.7±3.1	
MDS-MLD	16(20.0)	299.2±32.8		333.5±29.5		15.8(12.1–26.1)		4.8(4.1–11.3)		4.3(3.8–9.2)		89.6±3.3	
MDS-EB-1	20(25.0)	276.2±59.8	0.587	316.5±60	0.862	20.3(11.9–32.6)	0.005*	6.6(3.9–19.4)	0.004*	5.8(3.6–14)	0.004*	86.7±4.8	0.003*
MDS-EB-2	7(8.8)	270.4±81.7		319.3±82.8		24.7(20.7–27.8)		8.2(6.5–19.4)		7.0(5.8–14)		84.6±2.5	
Del(5q)	2(2.5)	305.4±8		355.9±18.9		25.2(21.4–29.1)		8.2(7.1–9.4)		7.1(6.2–7.9)		84.9±2.3	
Unclassifiable	5(6.3)	295.5±10.9		338.6±22.4		27.2(10.9–29.2)		9.3(3.7–9.7)		7.9(3.5–8.1)		84.7±2.7	
IPSS													
Low	35(43.2)	295.4±37.3		331.3±41.5		14.9(10.7–29.6)		5.8(3.7–10.6)		5.2(3.4–8.8)		89.1±3.4	
Int-1	37(46.3)	293.4±41.0	0.018*	332.0±40.7	0.085	16.4(11.5–32.6)	0.010*	6.4(3.9–13.4)	0.004*	5.6(3.6–10.5)	0.004*	88.2±3.5	<0.001*
Int-2	8(10.0)	245.8±88.5		291.9±89.2		24.1(14.7–27.8)		8.6(5.7–19.4)		7.3(5.1–14)		82.6±7	
R IPSS													
Very low	15(18.8)	289.5±52.0		325.3±56.1		15.6(10.9–29.6)		5.9(3.6–10.6)		5.3(3.4–8.8)		88.8±3.7	
Low	36(45.0)	304.7±24.4	0.005*	338.5±26.5	0.037*	14.9(10.7–29.2)	0.008	5.7(3.7–9.5)	0.001*	4.3(3.4–7.9)	0.001*	90.0±2.8	<0.001*
Intermediate	15(18.8)	294.1±42.3		334.5±43.2		16.4(11.9–28.8)		6.3(3.9–11.3)		5.7(3.6–9.2)		88.8±3.0	
Hıgh	12(15.0)	244.3±71.0		289.3±71.5		22.9(14.7–32.6)		9.1(5.5–19.4)		7.7(4.9–14)		83.4±6.1	
Cytogenetic													
Very good	3(3.7)	294.7±14.7		330.2±32.9		12.7(10.9–29.6)		4.2(3.9–9.7)		3.9(3.6–8.1)		89.6±5	
Good	73(91.3)	291.1±45.5	0.099	328.3±45.7	0.082	18(10.7–32.6)	0.48	6(3.6–19.4)	0.747	5.3(3.4–14)	0.747	88.4±3.9	0.187
Intermediate	3(3.7)	246±105.5		288.9±100.8		24.1(12.3–27.8)		9.3(3.9–19.4)		7.9(3.6–14)		83±10.4	
Very poor	1(1.3)	383.7		433.9		25.1		6.5		5.8		88.4	
Erythropoietin													
(+)	35(43.8)	289.2±54.8	0.809	323.1±54	0.411	15.1(10.7–29.6)	0.017*	4.8(3.6–19.4)	0.045*	4.4(3.4–14)	0.045*	89±4.6	0.151
(–)	45(56.2)	291.9±43.7		332.2±44.6		20.9(10.9–32.6)		6.7(3.7–19.4)		5.9(3.5–14)		87.7±4	
Hypometylating agent													
Azacitidine	27(33.8)	287.6±40.3		324.3±38.8		17.0(11.5–28.8)		6.1(3.6–11.3)		5.4(3.4–9.2)		88.5±3.4	
Decitabine	19(23.8)	286.8±73.3	0.765	328.3±71.5	0.863	20.8(12.1–32.6)	0.129	6.4(4.1–19.4)	0.249	5.7(3.8–14.0)	0.249	86.4±6.2	0.07
(–)	34(42.4)	295.4±37.3		331.3±41.5		14.8(10.7–29.6)		5.8(3.7–10.6)		5.2(3.4–8.8)		89.7±3.4	
Imid-treatment													
(+)	3(3.8)	303.3±71.4	0.649	345.9±76.8	0.525	22.5(16.3–25.1)	0.303	6.5(5.8–9.1)	0.514	5.8(5.2–7.7)	0.514	87.5±2.6	0.763
(–)	77(96.3)	290.2±48.1		327.5±48.1		18(10.7–32.6)		6(3.6–19.4)		5.3(3.4–14)		88.3±4.4	
Immunosuppressive treatment													
(+)	9(11.3)	283.7±26	0.650	322±25.2	0.686	19.5(12.7–27.8)	0.79	6.5(4.2–9.3)	0.626	5.8(3.8–7.9)	0.626	88.1±3.1	0.891
(–)	71(88.7)	291.6±50.7		329±51.1		18(10.7–32.6)		6(3.6–19.4)		5.3(3.4–14)		88.3±4.4	

The IMA levels of the MDS patients did not show any significant correlations with the demographic findings, clinical findings, or laboratory findings. Native thiol level was positively correlated with albumin (r=0.348; p=0.002) and negatively correlated with platelet level (r=0.300; p=0007); a negative correlation was found between IPSS score (r=-0.308; p=0.042), R-IPSS score (r=-0.306; p=0.044), and BM blasts (r=-0.300; p=0.026). The total thiol level was positively correlated with albumin (r=0.328; p=0.003) and platelet level (r=0.310; p=0.005) and negatively correlated with BM blasts (r=-0.308; p=0.048). Disulfide level was positively correlated with IPSS score (r=0.399; p<0.001) and R-IPSS score (r=0.331; p=0.003) and negatively correlated with neutrophil count (r= -0.306; p=0.026), hemoglobin level (r=-0.307; p=0.018), and BM blasts (r=-0.300; p=0.026). The disulfide/native thiol ratio was positively correlated with the IPSS score (r=0.506; p<0.001), R-IPSS score (r=0.413; p<0.001), and BM blast level (r=0.384; p<0.001) and negatively correlated with hemoglobin level (r=-0.295; p=0.036). The disulfide/total thiol ratio was positively correlated with IPSS score (r=0.501; p<0.001), R-IPSS score (r=0.417; p<0.001), and BM blast level (r=0.383; p<0.001) and negatively correlated with hemoglobin level (r=-0.296; p=0.030). The disulfide/total thiol ratio was positively correlated with hemoglobin level (r= 0.308; p=0.016) and negatively correlated with IPSS score (r=-0.407; p<0.001), R-IPSS score (r=-0.347; p<0.001), and BM blast level (r=-0.330; p<0.001).

The potential risk factors related to progression to AML are presented in Table 3. These potential risk factors were determined as hypertension, collagen tissue disease, MDS type, IPSS score, R-IPSS score, BM blasts, native thiol, disulfide/native thiol, disulfide/total thiol, native thiol/total thiol, hemoglobin level, and platelet level. In the multivariate Cox regression model, in which these potential risk factors were included, collagen tissue disease (HR: 9.17; p=0.005), MDS-EB-1 (HR: 10.14; p=0.032), MDS-EB-2 (HR: 18.2; p=0.043), and disulfide/native thiol (HR: 1.17; p=0.023) were found as independent predictors anticipating AML. Patients with collagen tissue disease were found to be 9.17 times more likely to develop AML than those without this disease. While MDS-EB-1 patients had 10.14 times higher risk of AML development compared to lower risk types, it was found that this risk was 18.2 times higher in MDS patients with MDS-EB-2. It was found that a 1% increase in the disulfide/native thiol ratio increased the risk of AML development 1.17 times (Table 5).

**Table 3:** Possible risk factors associated with the development of AML Parameters were expressed as mean ± SD and median (interquartile range), *P < 0.05 is considered significant for statistical analysis. *Abbreviations*: CVE: Cerebrovascular Event, COPD: Chronic Obstructive Pulmonary Disease, MDS: Myelodysplastic Syndrome, IPSS:International Prognosis Scoring System, R-IPSS: Revised International Prognosis Scoring System, IMA: Ischemia Modified Albumin, WBC:White Blood Cell

Variables	AML		Univariable		
	(–)	(+)	HR	95% CI	p
Age, (Year)	66.2±10.7	67.7±13.9	1.01	0.97–1.05	0.709
Gender, (%)					
Female	23(38.3)	5(25.0)	ref		
Male	37(61.7)	15(75.0)	1.55	0.56–4.27	0.397
Cancer, (%)	2(3.3)	3(15.0)	4.70	1.35–16.32	0.015*
Diabetes Mellitus, (%)	17(28.3)	8(40.0)	1.55	0.63–3.85	0.344
Hypertension, (%)	34(56.7)	18(90.0)	6.37	1.47–27.65	0.014*
Cardiac disease, (%)	14(23.3)	12(60.0)	3.05	1.25–7.45	0.015*
CVE, (%)	2(3.3)	2(10.0)	3.53	0.80–15.53	0.095
COPD, (%)	8(13.3)	3(15.0)	1.44	0.42–4.97	0.568
Renal disease, (%)	6(10.0)	2(10.0)	1.45	0.33–6.34	0.623
Thyroid disease, (%)	11(18.3)	–	0.04	0.01–9.90	0.251
Collagen Tissue Disease, (%)	1(1.7)	3(15.0)	8.71	2.29–33.11	
	0.001* MDS Type, (%)				
Low risk types	34(56.7)	3(15.0)	ref		
MDS-MLD	15(25.0)	1(5.0)	0.10	0.01–17.50	0.956
MDS-EB-1	9(15.0)	11(55.0)	11.56	2.56–52.18	0.001*
MDS-EB-2	2(3.3)	5(25.0)	35.34	6.71–186.04	<0.001*
IPSS score	0(0–1.5)	1(0–2)	9.23	4.0–21.28	<0.001*
R-IPSS score	2(1–5.5)	4.5(1–8.5)	2.05	1.55–2.70	<0.001*
IPSS category (%)					
Low	34(56.7)	–	ref		
Int-1	26(43.3)	11(55.0)	3.76	1.05–13.55	0.043*
Int-2	2(3.3)	6(30.0)	30.84	7.33–129.81	<0.001*
R-IPSS category (%)					
Very low	14(23.3)	1(5.0)	ref		
Low	31(51.7)	5(25.0)	2.13	0.2–18.41	0.493
Intermediate	9(15.0)	6(30.0)	5.68	0.67–48.42	0.112
Hıgh	6(10.0)	6(30.0)	13.21	1.54–113.10	0.018*
Very hıgh	–	2(10.0)	185.41	13.07–2630.9	<0.001*
Cytogenetic (%)					
Very good & Good	58(96.7)	18(90.0)	ref		
Non-good	2(3.3)	2(10.0)	2.98	0.68–13.01	0.147
K.I. Blast (%)	1.5(1–13)	6.0(2–17)	1.32	1.20–1.45	<0.001*
Albumın (g/L)	43±3	42±2	3.3	0.6–19.5	2.21
IMA (ng/mL)	0.71±0.09	0.71±0.05	0.45	0.01–135.47	0.783
Native thiol (μmol/L)	295.6±39.9	276.1±67.5	0.98	0.97–0.99	0.035*
Total thiol (μmol/L)	332.2±41.8	316.2±65.6	0.97	0.97–1.00	0.092
Disulfide (μmol/L)	16(10.7–32.6)	20.3(12.8–28.8)	1.05	0.98–1.13	0.169
SS/SH (%)	5.8(3.7–13.4)	6.6(3.6–19.4)	1.23	1.09–1.40	0.001*
SS/TT (%)	5.2(3.4–10.5)	5.8(3.4–14)	1.34	1.10–1.62	0.003*
SH/TT,(%)	88.9±3.6	86.4±5.7	0.86	0.78–0.95	0.003*
WBC (x10^3^ μL)	4160(1300–11600)	3535(1630–22000)	1.01	0.98–1.10	0.574
Neutrophile (x10^3^ μL)	2145(200–8100)	1345(220–9450)	1.10	0.90–1.05	0.206
Lymphocyte (x10^3^ μL)	1400(430–3320)	1910(600–2750)	1.00	0.99–1.01	0.086
Hemoglobin (g/L)	99±18	88±15	7.2	5.5–9.4	0.16*
Platelet (x10^3^ μL)	136.5(11–626)	86(10–271)	0.97	0.95–0.99	0.019*

The risk factors related to mortality are presented in Table 4. These potential risk factors were found to be age, diabetes mellitus, hypertension, cardiac disease, MDS type, IPSS score, R-IPSS score, BM blast level, native thiol, disulfide, disulfide/native thiol, disulfide/total thiol, native thiol/total thiol, hemoglobin level, platelet level, and AML presence. In the multivariate Cox regression model, in which these potential risk factors were included, age (HR: 1.05; p=0.002), MDS-EB-1 (HR: 12.58; p<0.001), MDS-EB-2 (HR: 5.75; p=0.033), disulfide/native thiol (%) (HR: 1.14; p=0.040), and hemoglobin (HR: 0.64; p=0.007) were found as independent predic-tors for mortality. A one-year increase in age caused mortality risk to increase 1.05 times. While the mortality risk of MDS patients with MDS-EB-1 was 12.58 times higher compared to patients with lower-risk types of MDS, it was found that in MDS patients with MDS-EB-2 mortality risk was 5.75 times higher. It was found that a 1% increase in the disulfide/native thiol ratio increased the mortality development risk by 1.14 times. It was also found that a 10 g/L decrease in hemoglobin level increased mortality risk by 1.56 times (1/0.64) (Table 5).

**Table 4:** Possible risk factors associated with mortality Parameters were expressed as mean ± SD and median (interquartile range), *P< 0.05 is considered significant for statistical analysis. *Abbreviation*s: CVE, Cerebrovascular Event; COPD, Chronic Obstructive Pulmonary Disease; MDS, Myelodysplastic Syndrome; IPSS, International Prognosis Scoring System; R-IPSS, Revised International Prognosis Scoring System; IMA, Ischemia Modified Albumin; AML, Acute Myeloid Leukemia; WBC, White Blood Cell.

Variables	Survival		Univariable		
	Alive	Exitus	HR	95% CI	p
Age (Year)	63.1±10.9	72.3±10.4	1.07	1.03–1.11	0.001*
Gender (%)					
Female	21(42.0)	7(23.3)	ref		
Male	29(58.0)	23(76.7)	2.00	0.85–4.69	0.110
Cancer (%)	2(4.0)	3(10.0)	2.54	0.76–8.55	0.131
Diabetes mellitus (%)	12(24.0)	13(43.3)	2.25	1.07–4.73	0.032*
Hypertension (%)	26(52.0)	26(86.7)	5.15	1.78–14.90	0.003*
Cardiac disease (%)	7(14.0)	19(63.3)	4.06	1.92–8.55	<0.001*
CVE (%)	1(2.0)	3(10.0)	2.97	0.88–9.93	0.078
COPD (%)	7(14.0)	4(13.3)	0.84	0.29–2.43	0.746
Renal disease (%)	4(8.0)	4(13.3)	2.53	0.85–7.53	0.096
Thyroid disease (%)	9(18.0)	2(6.7)	0.45	0.11–1.89	0.275
Collagen Tissue Disease (%)	2(4.0)	2(6.7)	1.63	0.38–6.91	0.510
MDS Type (%)					
Low risk type	34(68.0)	3(10.0)	ref		
MDS-MLD	10(20.0)	6(20.0)	3.60	0.86–15.11	0.080
MDS-EB-1	4(8.0)	16(53.3)	16.94	4.90–5.60	<0.001*
MDS_EB-2	2(4.0)	5(16.7)	20.53	4.81–87.66	<0.001*
IPSS score (%)	0(0–1.5)	1(0–2)	6.97	3.78–12.87	<0.001*
R-IPSS score (%)	2(1–5)	4.5(1–8.5)	2.07	1.67–2.56	<0.001*
IPSS category (%)					
Low	33(66.0)	1(3.3)	ref		
Int-1	17(34.0)	20(66.7)	8.56	2.54–28.86	0.001*
Int-2	1(2.0)	7(23.3)	47.58	11.06–204.70	<0.001*
R-IPSS category (%)					
Very low	14(28.0)	1(3.3)	ref		
Low	29(58.0)	7(23.3)	2.80	0.34–22.80	0.338
Intermediate	6(12.0)	9(30.0)	9.33	1.17–74.32	0.035*
Hıgh	1(2.0)	11(36.7)	75.14	8.90–634.72	<0.001*
Very hıgh	–	2(6.7)	151.83	11.85–	<0.001*
Cytogenetic (%)					
Very good & Good	48(96.0)	28(93.3)	ref		
Non-good	2(4.0)	2(6.7)	1.49	0.35–6.31	0.585
K.I. Blast (%)	1.0(1–13)	5(1–17)	1.21	1.12–1.30	<0.001*
AML (%)	2(4.0)	18(60.0)	4.97	2.38–10.40	<0.001*
Albumın (g/L)	43±3	42±2	4.5	1.0–19.8	2.89
IMA (ng/mL)	0.71±0.09	0.73±0.05	17.04	0.12–2491.5	0.265
Native thiol (mmol/L)	297.9±31.3	278.7±67.3	0.98	0.97–0.99	0.024*
Total thiol (mmol/L)	333.7±35.3	319.1±65.3	0.99	0.98–1.01	0.107
Disulfide, (mmol/L)	14.9(10.7–29.6)	20.6(12.8–32.6)	1.07	1.01–1.14	0.031*
SS/SH (%)	5.7(3.7–10.6)	6.6(3.6–19.4)	1.22	1.11–1.35	<0.001*
SS/TT (%)	5.1(3.4–8.8)	5.8(3.4–14)	1.34	1.15–1.56	<0.001*
SH/TT (%)	89.3±3.3	86.6±5.2	0.86	0.80–0.93	<0.001*
WBC (x103 μL)	4450(1300–11100)	3325(1740–22000)	1.00	0.99–1.01	0.720
Neutrophile (x103 μL)	2240(200–8100)	1495(220–9450)	1.05	0.99–1.10	0.070
Lymphocyte (x103 μL)	1470(430–3320)	1370(600–2750)	1.04	0.99–1.01	0.811
Hemoglobin (g/L)	103±18	86±13	5.3	4.0–7.0	<0.01*
Platelet (x103 μL)	171(19–626)	81(10–282)	0.95	0.90–0.99	0.005*

**Table 5:** Independent factors associated with AML and mortality *P< 0.05 is considered significant for statistical analysis. *Abbreviations*: AML: Acute Myeloid Leukemia, MDS: Myelodysplastic Syndrom

Variables	HR	95% CI	p
AML			
Collagen tissue disease MDS type	9.17	1.94– 43.30	0.005*
Low risk types	ref		
MDS-MLD	0.10	0.01– 43.19	0.951
MDS-EB-1	10.14	1.22–64.43	0.032*
MDS-EB-2	18.20	1.15–67.6	0.043*
SS/SH	1.17	1.02–1.34	0.023*
	-2Log Likelihood = 103.02; p<0.001		
Mortality			
Age MDS type	1.05	1.02–1.09	0.002*
Low risk types	ref		
MDS-MLD	2.75	0.64–11.74	0.173
MDS-EB-1	12.58	3.41– 46.48	<0.001*
MDS-EB-2	5.75	1.15–28.78	0.033*
SS/SH	1.14	1.01–1.29	0.040*
Hemoglobin	0.64	0.46–0.88	0.007*
	-2Log Likelihood = 167.15; p<0.001		

Disulfide/native thiol ratio of 5.9% was found as the optimal prediction point in anticipating progression to AML with 75% sensitivity and 55% specificity (+PV: 35.7%; -PV: 86.8%; AUC±SE: 0.640±0.07; p=0.043). Disulfide/native thiol ratio of 6.3% was found as the optimal prediction point in anticipating AML with 66.7% sensitivity and 64% specificity (+PV: 52.6%; -PV: 76.2%; AUC±SE: 0.669±0.06; p=0.006).

## Discussion

There are several studies reporting that disruptions in the apoptosis pathways and oxidative stress damage could play a role in the etiopathogenesis of MDS. However, we could not find any studies about the importance of dynamic thiol/disulfide homeostasis in MDS prognosis. Therefore, this is the first study conducted on this subject.

Oxidative stress and genetic instability in hema topoietic stem cell and myeloid progenitors are related to myeloid malignancy. The aging of hemato poietic cells, caused by both endogenic and exogenic cellular factors, disrupted hematopoietic environments, self-restoration, and regeneration, is related to immune system reconfiguration and the increase in leukemia incidence. Ghoti et al. [14] reported oxidative stress in erythrocytes, thrombocytes, and polymorphonuclear leukocytes and increased serum ROS levels in low-risk MDS patients. In addition, in another study, it was shown that this oxidative situation is related to the significant increase in oxidative DNA damage in BM cells in MDS patients. It is known that cancer cells adapt to oxidative conditions by elevating their antioxidant capacities, decreasing the ability to regulate other changes in oxidative stress compared to normal cells. The elevation of antioxidants as an adaptation to intracellular oxidative stress is a well-known phenomenon [14] [15].

The patient and control groups in our study had similar characteristics in terms of age and sex. When the data in the literature are reviewed, it is seen that total thiol, native thiol, and disulfide levels were shown to not have direct relations with many demographic characteristics such as hypertension, diabetes mellitus, cardiovascular disease, and age. The sulf hydryl groups of thiol compounds play an important role in the cellular defence against increasing free oxygen radicals. The increase of intracellular free oxygen radicals due to the decrease in thiol levels causes disruptions in the intracellular pathways and the apoptosis process. Erel and Neselioglu [10] showed that plasma disulfide levels are higher in patients with malignant and inflammatory diseases. In our study, it was observed that plasma disulfide was high in patients who previously had malignant diseases. It was also found that the thiol level was significantly lower in this study group. In a study on patients with multiple myeloma, it was found that the total thiol levels of the patients were significantly lower than those of the control group. In this respect, the results of our study showed parallelism with the findings in the literature [16] [17] [18].

It was found that the native thiol and total thiol levels were lower in MDS patients than the control group, and the disulfhydryl level was higher. While the increase in native thiol levels caused proliferation, it was shown that its decrease, together with the increase in disulfide, caused cell apoptosis. Although it was seen in previous studies with diabetes mellitus, hypertension, collagen tissue diseases, and cardiovascular diseases that total thiol, native thiol, and disulfide levels were higher in healthy individuals, previous studies on many cancer types showed that total and native thiol were significantly lower in cancer patients compared to healthy individuals. It is seen that high ROS levels reduce the self-restoration capacities of hematopoietic stem cells. The finding of thiol/disulfide imbalance, signified by thiol increase in our study, shows parallelism to the data provided in previous studies conducted with the method defined by Erel and Neselioglu [10]. It is expected that the disulfide level will increase as the thiol level decreases. These results suggest that a reduction in antioxidant enzyme activity could be a response to increasing ROS production, and, in time, the antioxidant enzyme activity could be insufficient for the detoxification of the high ROS levels. In a study on advanced-stage non-smallcell lung cancer, it was found that native thiol and total thiol were low, and it was argued that this decrease could be a prognostic marker for tumour aggression and malignant disease. In another study examining the relation between thiol/disulfide homeostasis in patients with advanced-stage colorectal carcinoma and clinical symptoms, it was found that elevated carcinoembryonic antigen and Ca 19.9 levels were notably related to lower native thiol and total thiol. The results of our study similarly showed that the increase in BM blasts and the increase in IPSS and R-IPSS scores, which are prognostic markers related to these parameters, were positively correlated with elevated disulfide and negatively correlated with total thiol. Our findings were thus compatible with the results of the studies conducted with patients with colon cancer and lung cancer [19] [20] [21].

IPSS and R-IPSS scores are determined as the most significant variables in studies on prognostic evaluation of patients with MDS and they are included in current guidelines. However, evaluation of both IPSS and R-IPSS depends on BM examination, hemogram findings, and cytogenetic analysis results. In our study, advanced age, high IPSS and R-IPSS scores, elevated BM blast ratio, and low hemoglobin and platelet values were found to be factors affecting mortality. In addition, patients' low levels of total thiol and native thiol and high levels of disulfide were found as negative prognostic markers. Peddie et al. argued that the highly oxidative conditions observed in progenitor cells such as blasts and erythroid precursors could be related to the increased oxidative DNA damage in CD34 cells [22]. The risk of secondary relapse of MDS is expected to be higher in patients with previous malignant diseases. Patients with high BM blast rates, low hemoglobin and thrombocyte levels, and increased IPSS and R-IPSS scores are at high risk of AML transformation. In addition, high disulfide levels and low total thiol levels are also anticipated to statistically predict AML risk. Previous studies with patients with malignities showed that high disulfide levels signalled a negative prognosis and high total thiol and native thiol exhibited protective characteristics [13]. Thus, low native thiol and high disulfide levels in MDS patients and the correlation of thiol/disulfide homeostasis with previously proven prognostic markers suggest that this could be a parameter that should be considered more carefully with regard to transformation to AML [13] [23].

The most significant limitation of our study was that the number of patients was small. In addition to the limited number of patients, the differences between the numbers of patients with different subtypes of MDS was another limitation. However, a control group was formed to provide a comparison. Another limitation was that we did not have a subsequent follow-up of the total thiol, native thiol, and disulfide levels for the MDS patients receiving treatment. However, we believe that the findings at the time of diagnosis are sufficient for etiopathogenesis and prognosis markers since these findings were significantly higher both among the patients and when compared to the control group; however, further studies are needed to evaluate the response to the treatment.

In conclusion, according to the findings of this study, we can argue that dynamic thiol/disulfide homeostasis has an important effect on both the etiopathogenesis and the survival of patients with MDS, and thiol/disulfide homeostasis could be included in new prognostic scorings. It could also be considered in risk assessment in MDS and as a mortality risk factor in the general treatment plans of patients. With multi-centre prospective and randomized controlled studies in the future, we believe that its importance will be better understood.

## Conflict of interest statement

The authors state that they have no conflicts of interest regarding the publication of this article.
